# Effects of variable intensity and constant intensity flywheel resistance training programs on specific soccer players’ performance

**DOI:** 10.3389/fphys.2024.1375438

**Published:** 2024-05-30

**Authors:** Pablo Asencio, Francisco J. Moreno, José Luis Hernández-Davó, Rafael Sabido

**Affiliations:** ^1^ Sports Research Centre, Department of Sport Sciences, Miguel Hernández University, Elche, Spain; ^2^ Faculty of Health Sciences, University Isabel I of Castilla, Burgos, Spain

**Keywords:** flywheel resistance training, soccer, training program, strength training, performance

## Abstract

Resistance training programs play a crucial role in optimizing soccer performance. The aim of this study is to compare performance outcomes in sport-specific tasks after implementing two different flywheel resistance training (FRT) programs: variable intensity (VI) and constant intensity (CI). Seventeen (*n* = 17) amateur footballers were divided into VI and CI groups with the same training volume. For the VI group, a decrease in inertial load was implemented every four sessions, whereas the CI group maintained a constant load during the entire program. After different familiarization sessions and testing (sprint, change of direction, jump, one-repetition maximum and flywheel strength variables), ten sessions of FRT were performed over 5 weeks. Both groups showed similar improvements in the one-repetition maximum (*p* < 0.01) but the CI group had significant improvements in the 10-m sprint (*p* = 0.04; ES = 0.72), emphasizing the potential benefits of medium inertial loads to maximize power and specificity in sport tasks. However, no significant differences were observed in the countermovement jump, change of direction and 30-m sprint, possibly attributed to neuromuscular fatigue from a high-volume training schedule and friendly matches. The study highlights the importance of considering training load distribution in FRT programs. The findings emphasize the need for complementary training to maximize the jump and change of direction abilities and caution against high-volume training and friendly match scenarios. In conclusion, FRT programs, whether varying in intensity or not, can yield medium-term performance improvements for soccer players.

## Introduction

Resistance training programs should consider various training variables over time (Kraemer and Ratamess, 2000) and there are different programming strategies to optimize the force–time relationship and consequently increase performance in sport-specific tasks (Suchomel et al., 2016). For this reason, programs should thoroughly control variables such as intensity, volume, density, frequency or exercise selection (Zatsiorsky et al., 2020). Training volume and training intensity have received the greatest attention in strength training programs. Intensity can be expressed as a percentage of the one-repetition maximum (1RM), velocity of the bar, repetitions in reserve or rating of perceived effort (RPE) (Suchomel et al., 2021). On the other hand, training volume is represented by the total session workload performed, which influences the magnitude of metabolic stress and muscle damage (Schoenfeld, 2010). Intensity and volume manipulation dictate the physiological and biomechanical resistance training demands. Therefore, a combination of training variables is essential for optimizing training outcomes, achieving fitness goals and reducing the risk of overtraining (Kraemer and Ratamess, 2004).

Over the last few years, flywheel resistance training (FRT) has gained a lot of relevance in the world of strength and conditioning (Maroto-Izquierdo et al., 2017). This technology involves the use of a rotating mass that stores and releases energy during exercise (de Keijzer et al., 2022). Furthermore, practitioners can maximize the training effect of flywheel resistance technology by generating greater eccentric than concentric power outputs, a phenomenon referred to as eccentric overload (Beato et al., 2020). In addition, flywheel devices offer the possibility of performing specific and multi-planar exercises, replicating sport actions (Raya-González et al., 2021). These characteristics have caused flywheel technology to be widely used in sports, most commonly in football (Beato et al., 2021). Research has proven the effectiveness of FRT in enhancing strength (Raya-González et al., 2021; Allen et al., 2023), power (Raya-González et al., 2021; Allen et al., 2023), change of direction (COD) (Coratella et al., 2019; Fiorilli et al., 2020), countermovement jump (CMJ) (Allen et al., 2023) and sprint performance (de Hoyo et al., 2015). In addition to the classical training variables, FRT requires the management of other variables, such as strap rewind height (Sabido et al., 2020), rope length (Sabido et al., 2020) and loading conditions (Asencio et al., 2022).

Despite the effectiveness of FRT at improving athletes’ performance, supported by previous research (Beato et al., 2021), only a few programming variables (mainly intensity/inertial load) have been studied in the scientific literature. This scarcity of research signifies a lack of knowledge about the optimal manipulation of basic variables during a FRT program. Most studies (de Hoyo et al., 2015; Sabido et al., 2018; Pecci et al., 2022) used a constant load approach (same inertial load during FRT), finding improvements in different performance variables such as the CMJ, sprint performance or 1RM squat. To the authors’ knowledge there is only one study (Sabido et al., 2017) that compared the effect of FRT on performance variables in rugby players who were divided into two training groups with different intensity during the intervention (0.075 kg m^2^ and 0.025 kg m^2^, respectively). The study reported 1RM squat and CMJ improvements in both groups but no improvement in COD and a possible decrease in sprint performance at 0.075 kg m^2^. However, for the 0.025 kg m^2^ group there were small changes in linear sprint and positive effects on the agility T-test. Recently, Beato et al. (2021) proposed methodological bases of flywheel periodization in team sports and the distribution of training variables along the microcycle. Nevertheless, there is a need to compare the effect between different types of FRT programs in medium- and long-term adaptations.

Due to the lack of research on the possible effects of different FRT programs, it is important to determine whether a change of training intensity over time is needed during an FRT program. The objective of this study is to compare two types of training programs in sport performance tasks: variable intensity (VI) and constant intensity (CI). We hypothesized that varying the intensity is necessary to achieve medium-term performance adaptations and, consequently, that the VI group will achieve higher levels of performance than the CI group.

## Materials and methods

### Study design

This experimental study was carried out during 7 weeks of the pre-season period (see Table 2). In Weeks 1 and 7, performance assessments were performed. After a familiarization procedure and testing, participants were divided into two groups differing in the type of intensity distribution (VI and CI). For the VI group, inertial load was changed from higher to lower (see Table 1), whereas for the CI group, the inertial load was constant during all training periods. Both groups trained with the same density and total volume.

**TABLE 1:** Training volume and intensity for the two groups. CI: constant intensity; VI: variable intensity; S: sets; R: repetitions; T: total repetitions.

				Squat			HORIZONTAL lunge (total sets)		
CI group	VI group			S	R	T	S	R	T
Inertia 0.08 kg*⋅m* ^*2*^	Inertia 0.12 kg*⋅m* ^*2*^	Week 1	*Session 1*	3	6	18	2	6	12
			*Session 2*	3	6	18	4	6	24
		Week 2	*Session 3*	4	6	24	4	6	24
			*Session 4*	4	7	28	4	6	24
	Inertia 0.10 kg*⋅m* ^*2*^	Week 3	*Session 5*	4	8	32	4	8	32
			*Session 6*	4	8	32	4	8	32
		Week 4	*Session 7*	4	8	32	4	8	32
			*Session 8*	4	8	32	4	8	32
	Inertia 0.08 kg*⋅m* ^*2*^	Week 5	*Session 9*	4	8	32	4	8	32
			*Session 10*	4	11	44	4	11	44
						292			288
T:									**580**

### Participants

Seventeen (*n* = 17) amateur footballers in the Spanish third division team took part in this study. Participants had at least 2 years of experience in resistance training. Previous power analysis was conducted to determine the appropriate sample size using G^∗^ Power (version 3.1.9.3, Dusseldorf, Germany). According to the study design (2 groups, 2 repeated measures), a medium effect size f = 0.8, a correlation between measurements of *r* = 0.6, an α = 0.05, a required power 1-β = 0.95, a sample of 16 participants was required (actual power = 0.95).

Participants were assessed for their 1RM and subsequently assigned to one of two homogeneous groups according to player’s role and strength level based on their 1RM/body mass (Haff and Triplett, 2021).

The VI group (*n* = 8; age = 22.00 ± 5.71 years; height = 1.82 ± 0.08 m; body mass = 76.20 ± 6.40 kg; 1RM = 132.48 ± 18.90; ratio 1RM/body mass = 1.74 ± 0.29) trained with decreasing inertial load every four training sessions (0.12 kg⋅m^2^; 0.10 kg⋅m^2^; 0.08 kg⋅m^2^) and the CI group (*n* = 9; age = 22.9 ± 7.2 years; height = 1.80 ± 0.04 m; body mass = 75.66 ± 6.13 kg; 1RM 130.41 ± 19.87 kg; ratio 1RM/body mass 1.80 ± 0.04) trained with 0.08 kg⋅m^2^ during the entire training period. Participants provided written informed consent in accordance with the Declaration of Helsinki and the study was approved by the ethics committee of the host institution (Code: ADH. DES.RSS.PAV.23).

### Procedures

**FIGURE 1:**
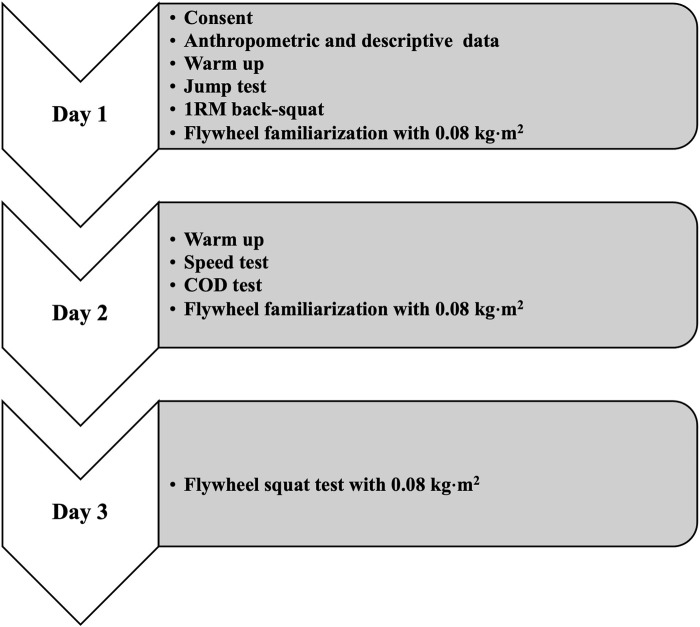
Scheme of the pretest.

During the first week, participants were tested on three separate sessions, with 72 h of recovery between sessions. On the first day, descriptive (e.g., age, training level) and anthropometric data were recorded for each participant. After that, jump tests and a 1RM back-squat test were performed and participants completed a flywheel familiarization protocol (Sabido et al., 2020). On the second day, participants conducted speed and COD tests and another flywheel familiarization protocol. On the last day, a flywheel squat exercise test was performed (see Figure 1).

### Testing session 1: CMJ and 1RM

Data on the CMJ and 1RM back-squat test were collected from the participants. A contact platform (Chronojump Boscosystem) was used to assess the CMJ. Participants were instructed to achieve their maximum jump height, with hands on their hips, and to execute the descending phase at their preferred depth. Three attempts were assessed and the best trial was used for analysis.

Data on the 1RM back-squat exercise were obtained using a linear encoder (T-Force System, Ergotech, Murcia, Spain) and a software application was used to calculate the relevant kinetic and kinematic parameters. For 1RM estimation, participants performed a protocol previously described by Loturco et al. (Loturco et al., 2016). Briefly, this consisted of starting from a shoulder-width stance with the barbell positioned on the upper back near the acromion and with the knees and hips fully extended. Each participant descended until the thighs were parallel to the ground and then they ascended to an upright position. Participants started with a load representing 50% of their body mass and thereafter the load was gradually increased until the mean propulsive velocity was <0.5 m s. Using this submaximal load, participants performed three maximal repetitions and with the linear position transducer attached it was possible to automatically estimate the 1RM of the athletes. A 4-min rest interval separated each test and the 1RM was estimated based on movement velocity, as previously described.

### Testing session 2: speed and COD

Acceleration, speed capacity and COD were evaluated on a grass soccer field. To assess speed, the 10-m sprint and 30-m sprint were performed. Participants stood 1 m behind the start line in a starting position with the body leaned forward. Timing gates (Microgate, Bolzano, Italy) were placed at the start (0 m), middle (10 m) and end (30 m), with reflectors at 1 m height (Tous-Fajardo et al., 2016). Participants were instructed to sprint at maximum speed for the entire distance. Each participant performed three attempts, with 2 min of passive recovery. The best score was used for the analyses.

COD was tested using the modified 505 test (M505), which involved two attempts of a 5-m sprint followed by a 180º COD and return to the starting point, which is a common maneuver in many sports (Raya-González et al., 2021). Timing gates (Microgate, Bolzano, Italy) were positioned at the starting and finishing points. Tests started on the “Go” command from a standing position, with the front foot 0.2 m from the photocell beam (Chaouachi et al., 2012).

### Testing session 3: flywheel squat

On the last testing day, participants completed a flywheel squat test with the flywheel device (VersaPulley, Iberian Sportech, Seville, Spain), carrying out a maximum set of eight repetitions, with an additional two initial repetitions needed to build momentum. The inertial load used during the test was 0.08 kg⋅m^2^. Participants performed two sets to warm up with a 2-min rest interval, a protocol recommended by Sabido et al. (2017). During each repetition the concentric and eccentric power (and their ratio) were recorded using a linear encoder and subsequently analyzed (SmartCoach Power Encoder, Europe AB, Stockholm, Sweden). The variables used for data analysis were peak concentric power (*PP*_*con*_), peak eccentric power (*PP*_*ecc*_) and the eccentric overload (EO) ratio.

### Training program

One week after the pre-test, participants started the training program using a flywheel device. Both groups engaged in two training sessions per week. The total volume of the training program was equated and the widest part of the conical pulley was chosen for setting the strap rewind height, aiming to maximize movement velocity (Sabido et al., 2020) (see Table 1). The program consisted of two exercises with different force vectors (vertical squat and horizontal lunge; see Table 1) twice a week. After a general warm up, each training session encompassed flywheel resistance exercises and a general soccer injury prevention program (e.g., core stability, balance and proprioceptive and hamstring eccentric exercises; see Table 2). During each set, two initial repetitions were needed to build inertia momentum and participants were instructed to perform each repetition as fast as possible and to delay braking action until the last third of the eccentric phase (Sabido et al., 2017). Rest intervals were standardized at 2 min, as specified by Sabido et al. (Sabido et al., 2018). The training protocols exhibited variations in training intensity, with a focus on either a conventional training block from high to low loads (G1) or constant load (G2) approaches (see Table 1). After Week 6, participants completed the post-test procedure.

**TABLE 2:** Weekly training plan for the training period. LSG: long side games; MSG: medium side games; SSG: small side games.

	Monday	Tuesday	Wednesday	Thursday	Friday	Saturday	Sunday
*Week 1*		Testing	Testing	Strength exercises technique LSG–MSG 2x (5vs5) + 3	Strength exercises technique LSG (11vs11) 3 × 12’	REST	REST
*Week 2*	AM: Testing PM: Flywheel training–Injury prevention SSG-MSG (7vs7) 4 × 8’	LSG (11vs11) 3 × 12’ Upper body strength work	AM: Flywheel training–Injury prevention PM: Friendly match	Upper body strength work	LSG–MSG 2 × 12’	Friendly match	REST
*Week 3*	AM: SSG–MSG 4 × 6’ PM: Flywheel training–Injury prevention	LSG–MSG 2 × 12’ Upper body strength work	AM: LSG (11vs11) 3 × 13’ PM: Flywheel training–Injury prevention	MSG - SSG	LSG–MSG 2 × 12’	Friendly match	REST
*Week 4*	LSG–MSG 4 × 6’ Flywheel training–Injury prevention	Friendly match	MSG–SSG Flywheel training–Injury prevention Upper body strength work	MSG 4 × 6’ (5vs5)	Friendly match	REST	REST
*Week 5*	SSG (4vs4)/(3vs3) 2 × 6 × 1’/1’ Flywheel training–Injury prevention	LSG–MSG Upper body strength work	MSG 4 × 6’ (5vs5) + 3 Flywheel training–Injury prevention	LSG–MSG Upper body strength work 2 × 12’	Friendly match	Friendly match	REST
*Week 6*	SSG (4vs4)/(3vs3) 2 × 6 × 1’/1’ Flywheel training–Injury prevention	LSG–MSG 2 × 12’ Upper body strength work	Friendly match	LSG–MSG Upper body strength work 2 × 12’	SSG (4vs4)/(3vs3) 2 × 6 × 1’/1’ Flywheel training–Injury prevention	Friendly match	REST
*Week 7*	Testing						

### Statistical analysis

Statistical analyses were performed using SPSS statistics package version 25.0 (IBM, New York, NY, United States of America). Following the size of the sample, confirmation of data normality using Shapiro-Wilk test was performed. Levene’s test for homogeneity of variances was employed to assess the equality of variances across groups or conditions. To assess the assumption of sphericity in repeated measures or within subjects, Mauchly’s sphericity test was employed. The effectiveness of each program (VI and CI) on the time was evaluated using a mixed model (time per group) ANOVA. A Bonferroni *post hoc* test for pairwise comparisons was conducted and the level of statistical significance was set at *p* < 0.05. Individual data analysis was presented using 2*SEM (Standard Error of the Mean) to establish individual changes between responders and non responders athletes. To assess the magnitude of the changes, Cohen’s d effect size (ES) calculation was performed, with interpretations as trivial (<0.2), small (0.2–0.5), moderate (0.5–0.8) and large (>0.8) (Ferguson, 2009).

## Results

After confirm data normality, Levene’s test indicated homogeneity of variances (*p* > 0.05). To present the results more precisely according to the Mauchly’s test, Greenhouse-Geisser criterion was selected to control Type I error rates.

The group factor showed that there were no significant differences between groups in initial conditions, indicating two homogeneous groups in all measured variables after balanced assignment.

No significant differences were observed in the time × group interaction (*p* > 0.05). The Time factor reported that there were pre-to post-changes in performance variables (see Table 3). Positive improvement in performance variables is shown by the positive values of effect size. Both groups showed no changes in CMJ height, M505-ND or 30-m sprint time. Furthermore, both groups showed significant decreases in M505-D.

**TABLE 3:** Changes in performance after the variable intensity (VI) and constant intensity (CI) programs. CMJ: countermovement jump; M505-D: modified 505-Dominant side; M505-ND: modified 505 non-dominant side; 1RM: one-repetition maximum; *PP*_*con*_: concentric peak power; *PP*_*ecc*_: eccentric peak power; EO: eccentric overload; %: percentage change; ES: effect size; CI: confidence interval; *: *p* < 0.05.

VI group (*n* = 8)						CI group (*n* = 9)				
Variable	Pretest	Posttest	ES	CI	%	Pretest	Posttest	ES	CI	%
CMJ (cm)	39.39 ± 4.29	37.94 ± 5.28	−0.30	(-0.58, −0,02)	−3.68	42.20 ± 5.30	41.57 ± 4.90	−0.11	(-0.33, 0.11)	−1.49
M505-D (s)	2.51 ± 0.06	2.61 ± 0.13*	−1.29	(0.07, 2.51)	3.98	2.49 ± 0.06	2.62 ± 0.09*	−1.83	(1.07, 2.60)	5.37
M505-ND (s)	2.58 ± 0.13	2.69 ± 0.23	−0.73	(-0.16, 1.62)	4.26	2.52 ± 0.06	2.62 ± 0.16	−1.32	(-0.42, 3.06)	3.79
SPRINT 10 m (s)	1.83 ± 0.09	1.80 ± 0.09	0.36	(-1.09, 0.36)	−2.06	1.81 ± 0.09	1.74 ± 0.07*	0.72	(-1.34, −0.11)	−4.02
SPRINT 30 m (s)	4.24 ± 0.17	4.30 ± 0.18	−0.31	(-0.09, 0.71)	1.41	4.16 ± 0.19	4.15 ± 0.16	0.05	(-0.38, 0.28)	−0.24
1RM (kg)	134.47 ± 20.84	141.12 ± 26.17*	0.28	(0.11, 0.46)	4.94	132.23 ± 20.41	140.68 ± 21.53*	0.37	(0.07, 0.68)	6.39
*PP*_*con*_ (W)	1488.21 ± 338.14	1628.54 ± 312.85	0.37	(-0.13, 0.87)	9.42	1635.11 ± 443.37	1808.24 ± 395.62	0.35	(0.08, 0.63)	10.50
*PP*_*ecc*_ (W)	1866.67 ± 531.74	2120.32 ± 573.33	0.42	(-0.08, 0.93)	13.58	1988.74 ± 448.68	2299.68 ± 465.61	0.63	(0.13, 1.12)	15.60
EO	25.18 ± 24.52	30.03 ± 19.13	0.18	(-0.43, 0.79)	19.26	29.68 ± 34.26	28.40 ± 15.42	−0.03	(-0.59, 0.53)	−4.31

However, in the 10-m sprint, the CI group showed significant improvements (*p* = 0.04). There were also significant improvements in the 1RM values for both groups (*p* < 0.01).

**FIGURE 2:**
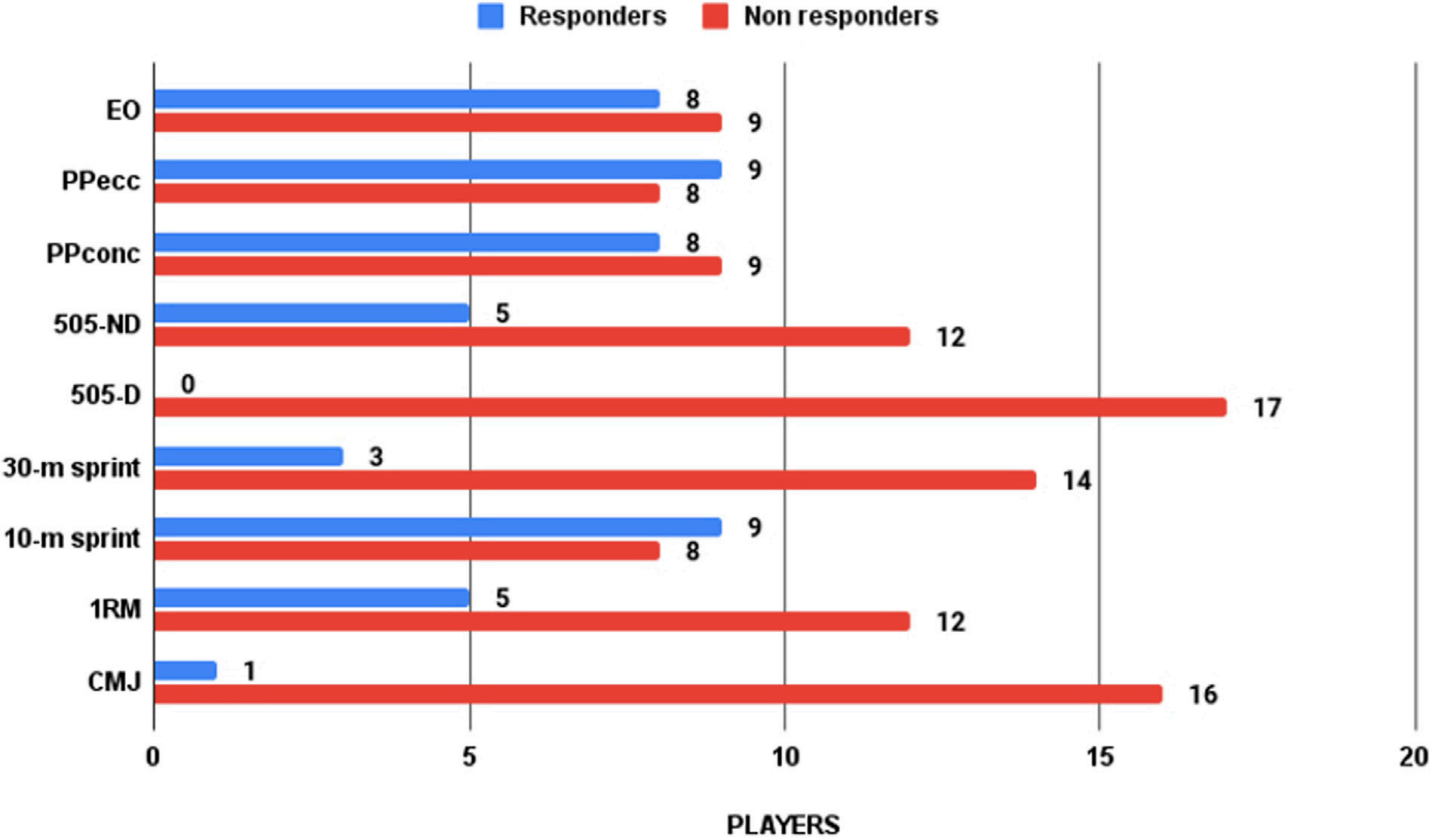
Individual results of different fitness testing. Athletes were divided into responders and non-responders based on 2xSEM criteria.

Finally, for the flywheel performance variables, both groups showed increases in *PP*_*con*_ (VI: 9.42%; CI: 10.50%) and *PP*_*ecc*_ (VI: 13.80%; CI: 15.60%). Due to small sample size, to confirm the results, individual target has been contrasted with an individual analysis using 2xSEM (see Figure 2).

## Discussion

This study aimed to compare the effects of VI and CI FRT programs on soccer players’ fitness performance. The main findings of this research are: the VI and CI groups have similar improvements in the 1RM variable; the CI group shows significant improvement for the 10-m sprint; and no difference was observed in the other variables apart from a significant decrease in M505-D.

Previous studies have reported the relationship of the 1RM squat with different performance tests in soccer (Requena et al., 2009; Comfort et al., 2014) and its influence on players’ performance (Owen et al., 2015; Wing et al., 2020). The benefits of FRT obtained in our study are similar to previous works (Fernandez-Gonzalo et al., 2014; Sabido et al., 2017; Sagelv et al., 2020). Thus, the inclusion of FRT can be an optimal way to improve maximal strength in lower limbs, optimizing sprinting (De Hoyo et al., 2016) and jumping (Wisløff et al., 2004), as a tool to improve match actions (Wing et al., 2020) or as an indicator of fatigue recovery after competition (Owen et al., 2015). The benefits mentioned for sprint performance have been observed in our results over short distances and are very important in soccer (Chelly et al., 2010). Individual outcomes surpassing twice the value of SEM for the 10-m sprint test and flywheel squat power values indicate substantial performance changes according to the previous statistical analysis. These results agree with previous studies using FRT (Núñez et al., 2018; Suarez-Arrones et al., 2018; Coratella et al., 2019; Sagelv et al., 2020; Raya-González et al., 2021). Although the trend to improve the 10-m sprint test is observed in both groups, only the CI group obtained a significant difference after training. This finding could be due to the CI group using lower inertial loads. These results are relevant because sprint ability is one of the most important performance variables in soccer (Castillo et al., 2020), being linked to soccer-specific tasks both in defensive and offensive actions (Mara et al., 2017; Cochrane and Monaghan, 2021). According to Sabido et al. (Sabido et al., 2018), lower inertial loads are a better option for eliciting high concentric peak power output values, and, according to our results, a low load where high power is produced can be the best choice to optimize short-sprint ability. For this reason, the increases in *PP*_*con*_ and *PP*_*ecc*_ are greater in the CI group compared to the VI group (10.50% vs. 9.42% and 15.60 vs. 13.58 in *PP*_*con*_ and *PP*_*ecc*_, respectively). Accordingly, recent research (Asencio et al., 2024) shows that lower inertial loads can be optimal for trained subjects to obtain the maximal power values in the concentric and eccentric phases during squat exercises in FRT.

Studies on FRT have reported that this methodology can be very useful for improving jumping ability, COD and sprint tasks in soccer players (Gonzalo-Skok et al., 2017; Coratella et al., 2019; Fiorilli et al., 2020). Nevertheless, our results show that the CMJ, COD and 30-m sprint did not improve in any of the groups after ten sessions of FRT, and even worse values were found for M505-D. These results are in line with individual analysis, showing a similar or high number of non-responders for several tests. Two reasons may explain the results obtained in our study. On the one hand, the absence of complementary training to improve jump ability has been proposed by Pecci et al. (Pecci et al., 2022), who also did not find significant differences with female soccer players in CMJ height after 6 weeks of FRT. Thus, complementary tasks must be combined with FRT to obtain possible benefits in jump or COD abilities. On the other hand, the main hypothesis for these results is neuromuscular fatigue due to the high number of friendly matches (Hernández-Davo et al., 2022). The purpose of investigating in an ecological context implied different changes in the FRT program and the impossibility of resting at 72 h from the last match to the final tests (De Hoyo et al., 2016; Romagnoli et al., 2016).

To the authors’ knowledge, this is the first study to compare the effects of two different FRT programs (VI and CI) on sports performance. Despite this, our study has some limitations. Firstly, the training volume and friendly matches calendar were very high, so it may not be possible to minimize post-match fatigue levels (Nédélec et al., 2012). Secondly, even though each player completed at least ninety percent of the sessions, the player role and external training load may be influencing the results. Thirdly, no control group was included because FRT was considered an important variable not only for optimizing strength in players but also for reducing the probability of injury. For this reason, and to have a greater number of players in each group, a control group was not considered in this study. Finally, after training protocol no anthropometrical mesurements (i.e., body mass) were recorded. As previous research shown, the values of post-test can be conditioned by anthropometric changes (Koźlenia et al., 2020; Popowczak et al., 2021).

The findings of this study have a number of practical implications. Present findings suggest that VI and CI training improved strength levels. In addition, CI group showed significant improvements in the 10-m sprint. However, probably another type of training load (e.g., lower intensity and volume or complementary training) is needed to maximize performance in specific tasks such as the CMJ or COD test. Furthermore, it is necessary to control the fatigue levels and friendly matches calendar in pre-season periods in order to achieve functional overreaching. Thus, strength and conditioning coaches of soccer players with high 1RM/body mass ratio should to individualize FRT programs using CI (medium and lower inertias) and perform complementary training in promoting specific soccer performance improvements. Due to competitive density of most team-sports with short pre-season periods, this study concludes how to optimize performance outcomes using FRT in short periods of time (i.e., ten sessions during 5 weeks).

## Data Availability

The original contributions presented in the study are included in the article/supplementary material, further inquiries can be directed to the corresponding author.
